# Association of dipeptidyl peptidase-4 inhibitor and recurrent pancreatitis risk among patients with type 2 diabetes: A retrospective cohort study

**DOI:** 10.3389/fphar.2024.1341363

**Published:** 2024-07-04

**Authors:** Yi-Sun Yang, Edy Kornelius, Yu-Hsun Wang, Shih-Chan Lo, Chien-Ning Huang

**Affiliations:** ^1^ School of Medicine, Chung Shan Medical University, Taichung, Taiwan; ^2^ Department of Internal Medicine, Division of Endocrinology and Metabolism, Chung Shan Medical University Hospital, Taichung, Taiwan; ^3^ Department of Medical Research, Chung Shan Medical University Hospital, Taichung, Taiwan

**Keywords:** recurrent pancreatitis, type 2 diabetes, dipeptidyl peptidase-4 inhibitor, retrospective study, anti-diabetic drugs

## Abstract

**Introduction:** Following the introduction of incretin-based drugs to the market, instances of acute pancreatitis have been reported, leading the FDA to mandate a warning label. Incretin-based therapy has been linked to a rare yet significant adverse event known as acute pancreatitis. However, these concerns of use of incretin therapy remained an ongoing debate.

**Methods:** This retrospective cohort study was extracted data from the National Health Insurance (NHI) program in Taiwan focused on those having prior hospitalization history of acute pancreatitis. We identified adult patients with type 2 diabetes, all patients who received new prescriptions one year after the diagnosis of hospitalization for acute pancreatitis for DPP-4 inhibitors (index date). Study participants were divided into two groups: those taking DPP-4 inhibitors (the DPP-4 inhibitors group, *n* = 331) and those not taking DPP-4 inhibitors (the non- DPP-4 inhibitors group, *n* = 918). The outcome of interest is the recurrence of hospitalization of acute pancreatitis.

**Results:** The incidence density (per 1000 person-years) of acute pancreatitis was 23.16 for DPP-4 inhibitors group and 19.88 for non-DPP-4 inhibitor group. The relative risk is 0.86 (95% confidence interval (CI) 0.53–1.38). Results from the Cox proportional hazard model (HR) analysis, the DPP-4 inhibitor was associated with a neutral risk of acute pancreatitis HR 0.68; 95% CI: 0.42–1.09.

**Conclusions:** In this extensive nationwide cohort study conducted in Taiwan, involving a substantial number of newly diagnosed cases, the utilization of DPP-4 inhibitors appears to show no significant correlation with an elevated risk of acute pancreatitis, even among diabetic patients deemed to be at a high risk. These results extend the safety reassurance of incretin-based therapy to individuals considered high-risk for such complications.

## Introduction

Dipeptidyl peptidase-4 (DPP-4) inhibitors constitute a class of medications employed in the treatment of type 2 diabetes (T2D). The first DPP-4 inhibitor, sitagliptin, was approved by the U.S. Food and Drug Administration (FDA) in 2006 for the treatment of T2D. The trend of use has been increasing worldwide (Deacon and Holst, 2013), including in Taiwan. They are generally well tolerated, weight neutral, and do not increase the risk of hypoglycemia (Fakhoury et al., 2010; Scheen, 2018). During clinical use, as monitored through post-marketing surveillance and in extensive studies evaluating long-term cardiovascular safety, no notable imbalances in safety signals were detected (Dore et al., 2009; Scirica et al., 2013; White et al., 2013; Green et al., 2015; Scheen, 2018; Rosenstock et al., 2019a; Rosenstock et al., 2019b). However, some signals of increased risk of pancreatitis and bullous pemphigoid were detected (Huang et al., 2020), but the occurrence of these adverse events is rare. The discussions regarding the potential risk of acute pancreatitis continue to be a subject of ongoing debate.

Observational studies examining the connection between pancreatitis and DPP-4 inhibitors, utilizing clinical databases from diverse countries, have produced conflicting outcomes (Chou et al., 2014; Yang et al., 2016; Pinto et al., 2018). Most of these studies either omitted patients exhibiting signs of acute pancreatitis or failed to incorporate an adequate number of individuals at a heightened risk of experiencing acute pancreatitis. Only one study has specifically investigated individuals with a history of prior hospitalization for acute pancreatitis or those with hypertriglyceridemia, both of whom are considered at a high risk of developing acute pancreatitis in patients with T2D (Chang et al., 2016). The study evaluated the risk of sitagliptin compared to that with acarbose (Chang et al., 2016). There was no significant association found between sitagliptin use and an elevated risk, with an adjusted hazard ratio (HR) of 0.95 and 95% confidence interval (95% CI) of 0.79–1.16. Similar outcomes were observed in subgroups, including patients with a history of prior hospitalization for acute pancreatitis and those with hypertriglyceridemia. However, the percentage of a prior hospitalization history for acute pancreatitis included in this study was only around 6%–8%. The majority of them were patients with hypertriglyceridemia receiving fibrates (83%). Therefore, in this study, we particularly focused on those having a prior hospitalization history for acute pancreatitis.

## Methods

### Dataset

We used Taiwan’s National Health Insurance (NHI) program database for this study analysis. Taiwan’s NHI program is a government-run, single-payer healthcare system. It was implemented in 1995 and is one of the most comprehensive and successful universal healthcare systems globally. It provides coverage to nearly the entire population of Taiwan, with participation rates close to 99.99% (Hsieh et al., 2019). The National Health Insurance Research Database (NHIRD) encompasses comprehensive records of outpatient visits, hospital admissions, prescriptions, illnesses, and vital status for 99% of the nation’s population. Diagnosis codes in the NHIRD were validated. The study cohort was derived through a random sample selection process from the entire diabetic population within the NHIRD. The Longitudinal Health Insurance Databases (LHIDs) were utilized, involving the random sampling of one million beneficiaries from the original NHIRD in 2000, 2005, and 2010, respectively. The LHIDs contain the most updated claim data of sampled individuals since 1997. This retrospective cohort study collected data from the NHI program in Taiwan spanning from January 2000 to December 2018. The study received approval from the Ethics Committee for clinical research at the Chung Shan Medical University Hospital.

### Study population

First, we identified adult patients with T2D, aged over 20 years, between 1 January 2001 and 31 December 2016, who had initiation of any anti-diabetic drugs for more than 1 year. Within this cohort, we further identified those who had diagnosis of acute pancreatitis (hospitalization) between 1 January 2001 and 31 December 2016. We excluded those who had recurrent pancreatitis within 1 year after the diagnosis of acute pancreatitis; those receiving either GLP-1 receptor agonists or DPP-4 inhibitor before diagnosis of acute pancreatitis and within 1 year after acute pancreatitis, chronic pancreatitis, and pancreatic cancer; and any missing data. The missing data are minimal; up to 99.99% of Taiwan’s population are enrolled under this program. The information contained in the NHIRD is stored in different datasheets, including the registry for beneficiaries, ambulatory care claims, inpatient claims, prescriptions dispensed at pharmacies, registry for medical facilities, and registry for board-certified specialists. Some missing codes would be possible to occur; therefore, we exclude missing data. From above inclusion and exclusion criteria, we identified 1,249 patients entered to the cohort study. Additionally, we isolated users of DPP-4 inhibitors and matched them in a 1:1 ratio with randomly selected participants without DPP-4 inhibitor usage based on the age, sex, drug index date, and propensity score. The DPP-4 inhibitor users were monitored from their initial prescription of DPP-4 inhibitors until the study event occurred or until the conclusion of the study. This study employed an intention-to-treat analysis, meaning that participants were analyzed based on their initially assigned group, irrespective of their adherence or duration of DPP-4 inhibitor usage.

### Medication

The approval for marketing DPP-4 inhibitors in Taiwan occurred in 2009, 2011, 2011, and 2012 for sitagliptin, saxagliptin, vildagliptin, and linagliptin, respectively. We identified all individuals who received new prescriptions 1 year following their hospitalization diagnosis for acute pancreatitis for DPP-4 inhibitors (index date), either as monotherapy or in combination with other medications. Additionally, information regarding the use of concurrent medications, such as insulin and other oral anti-diabetic agents, was gathered, with anatomical therapeutic chemical (ATC) codes provided in Supplementary Table S1.

### Outcome

The study cohort was tracked from the index date until the occurrence of hospitalization for acute pancreatitis, which is defined as having a discharge diagnosis of ICD-9-CM code 577.0 or ICD-10-CM code K85, or until death, disenrollment from the NHI, or the conclusion of the follow-up period (31 December 2018).

### Covariates and ICD-9-CM and ICD-10-CM codes

The disease codes are derived from the International Classification of Diseases, ninth revision, Clinical Modification (ICD-9-CM), and the 10th revision, CM (ICD-10-CM). Newly diagnosed T2D was defined as the first occurrence of a T2D code in outpatient records with at least three ambulatory claims or in one inpatient claim between January 2000 and December 2018. Acute pancreatitis was identified by the presence of a primary or secondary ICD-9-CM code of 577.0 or ICD-10-CM code K85 during hospitalization. The list of ICD-9 and ICD-10 codes used to define the inclusion of T2D, study outcome events, and comorbidities can be found in Supplementary Table S1.

### Statistical analysis

Baseline characteristics, comorbidities, and medication utilization among those who initiated DPP-4 inhibitors and those who did not were summarized. Data are expressed as valid percentages and mean values with standard deviations. The propensity score method was employed to assess the impact of the two study groups on the study outcomes. Person-days of follow-up were calculated for all participants in the two treatment groups. We computed the crude incidence rates for acute pancreatitis and estimated their 95% CI based on a Poisson distribution. A Cox proportional hazard regression model, stratified by baseline propensity score quintiles, was employed to determine the HR of hospitalization for acute pancreatitis, with non-DPP-4 inhibitors serving as the reference group, and their corresponding 95% CI. The time-to-event outcome is the time from a specific starting point (enrollment in a study and prescription of DPP-4 inhibitor) to the occurrence of acute pancreatitis. Additionally, univariate and *multivariate* cox proportional hazard models are used to evaluate the HR and 95% CI, adjusting for key risk factors associated with the development of study events, such as age, sex, medication use, and comorbidities. All analyses were conducted utilizing an intention-to-treat approach. Statistical significance was established at a *p*-value less than 0.05. The data were analyzed using IBM SPSS Statistics (version 27).

## Results

**FIGURE 1:**
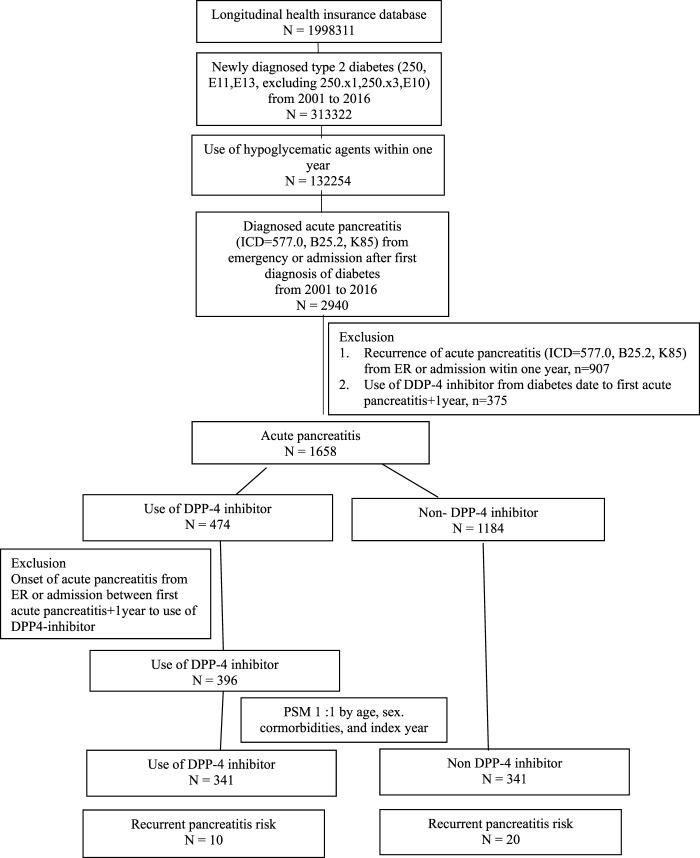
Design and flowchart of this retrospective cohort study.

**FIGURE 2:**
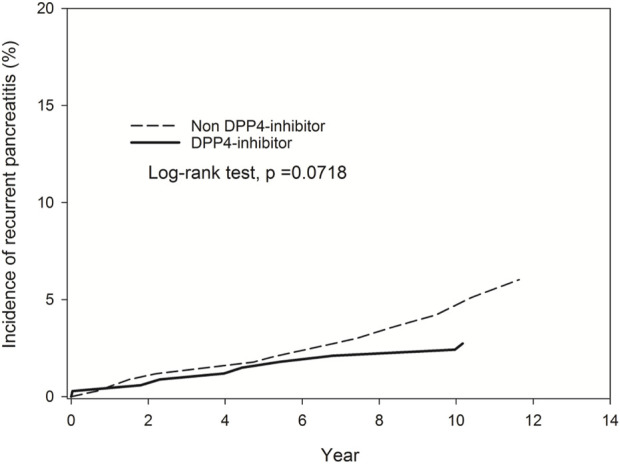
Kaplan–Meier curves for time to hospitalization for acute pancreatitis did not differ in patients on DPP-4 inhibitors and those on non-DPP-4 inhibitors.

The study participants were categorized into two groups: individuals using DPP-4 inhibitors (referred to as the DPP-4 inhibitors group, n = 331) and those not utilizing DPP-4 inhibitors (designated as the non-DPP-4 inhibitors group, n = 918). Within the DPP-4 inhibitors group, 58 patients were excluded due to a pre-existing diagnosis of pancreatitis before the index date. Consequently, a total of 273 patients were included in the DPP-4 inhibitors group. A flowchart for the enrollment of the study cohort is summarized in Figure 1. Table 1 illustrates the baseline characteristics of all patients in both the DPP-4 inhibitors group and the non-DPP-4 inhibitors group. The two treatment cohorts exhibited similarities in the majority of baseline characteristics, encompassing pre-existing comorbidities and medical histories such as cholelithiasis, pure hypertriglyceridemia, and alcoholic liver disease, all recognized as risk factors for acute pancreatitis. Nonetheless, a higher percentage of initiators of DPP-4 inhibitors also received other oral anti-diabetic drugs. A total of 20 cases in the DPP-4 inhibitors group and 111 cases in the non-DPP-4 inhibitor group were hospitalized for acute pancreatitis during the follow-up period. As shown in Table 2, the incidence density (per 1,000 person-years) of acute pancreatitis was 23.16 for the DPP-4 inhibitors group and 19.88 for the non-DPP-4 inhibitor group. The relative risk (RR) is 0.86 (95% CI: 0.53–1.38). The Kaplan–Meier curves for time to hospitalization for acute pancreatitis did not differ in patients on DPP-4 inhibitors and those on non-DPP-4 inhibitors (Figure 2). Table 3 presents results from the Cox proportional hazard model analysis. With non-DPP-4 inhibitors as the reference group, in univariate analysis, the DPP-4 inhibitor was associated with a neutral risk of acute pancreatitis (HR: 0.68; 95% CI: 0.42–1.09). After controlling for age, sex, comorbidities, and anti-diabetic drugs, in multivariate analysis, the DPP-4 inhibitors were associated with a neutral risk of acute pancreatitis (HR: 0.63; 95% CI: 0.38–1.07). Among other anti-diabetic drugs, metformin, thiazolidinediones, alpha-glucosidase inhibitors, sulfonylureas/glinides, and insulin did not reach statistical significance for occurrence of acute pancreatitis, as shown in Table 3.

**TABLE 1:** Demographic characteristics of DPP-4 inhibitor and non-DPP-4 inhibitor.

	Before PSM matching				*p*-value	After PSM matching				*p*-value
	Non-DPP-4 i (N = 1,184)		DPP-4 i (N = 396)			Non-DPP-4 i (N = 341)		DPP-4 i (N = 341)		
	n	%	N	%		N	%	n	%	
Age (years)					0.0014					0.5875
<40	104	8.8	22	5.6		16	4.7	20	5.9	
40–64	542	45.8	221	55.8		200	58.7	188	55.1	
≥65	538	45.4	153	38.6		125	36.7	133	39.0	
Mean ± SD	61.70 ± 16.11		61.42 ± 13.70		0.7412	60.90 ± 14.88		61.28 ± 13.60		0.7271
Sex					0.3137					0.6952
Female	430	36.3	155	39.1		132	38.7	137	40.2	
Male	754	63.7	241	60.9		209	61.3	204	59.8	
Biguanides	499	42.1	264	66.7	<.0001	171	50.1	230	67.4	<.0001
Glinides	96	8.1	61	15.4	<.0001	27	7.9	52	15.2	0.0028
Alpha-glucosidase inhibitors	86	7.3	92	23.2	<.0001	26	7.6	78	22.9	<.0001
Sulfonylurea	452	38.2	284	71.7	<.0001	125	36.7	245	71.8	<.0001
Thiazolidinediones	60	5.1	82	20.7	<.0001	24	7.0	71	20.8	<.0001
Insulin	639	54.0	149	37.6	<.0001	181	53.1	130	38.1	<.0001
Hypertension	569	48.1	241	60.9	<.0001	200	58.7	201	58.9	0.9380
Hyperlipidemia	318	26.9	205	51.8	<.0001	160	46.9	170	49.9	0.4435
Chronic liver disease	338	28.5	67	16.9	<.0001	61	17.9	57	16.7	0.6855
Chronic kidney disease	95	8.0	41	10.4	0.1524	32	9.4	32	9.4	1.0000
COPD	109	9.2	27	6.8	0.143	20	5.9	24	7.0	0.5330
Malignancy	185	15.6	32	8.1	0.000	22	6.5	29	8.5	0.3082
Intracranial bleeding	19	1.6	4	1.0	0.392	5	1.5	4	1.2	0.7372
Stroke/TIA	122	10.3	26	6.6	0.027	24	7.0	25	7.3	0.8821
Ischemic heart disease	176	14.9	58	14.6	0.916	47	13.8	51	15.0	0.6624
ER/admission diagnosis										
Cholelithiasis	168	14.2	67	16.9	0.186	58	17.0	58	17.0	1.0000
Alcoholic liver disease	53	4.5	8	2.0	0.028	5	1.5	7	2.1	0.5602
Pure hyperglyceridemia	18	1.5	9	2.3	0.317	4	1.2	7	2.1	0.3618

SD, standard deviation; TIA, transient ischemic stroke; COPD, chronic obstructive pulmonary disease; ER, emergency room.

**TABLE 2:** Poisson regression of relative risk of non-DPP-4 i and DPP-4 i.

	Non-DPP-4 inhibitor	DPP-4 inhibitor
N	341	341
Person-years	3,941	3,908
No. of acute pancreatitis	20	10
ID (95% CI)	5.07 (3.27–7.87)	2.56 (1.38–4.76)
Relative risk (95% CI)	Reference	0.50 (0.24–1.08)

ID, incidence density (per 1,000 person-years).

CI, confidence interval.

**TABLE 3:** Cox proportional hazard model analysis for risk of acute pancreatitis.

	Univariate	*p*-value	Multivariate	*p*-value
	HR (95% CI)		HR (95% CI)	
Group				
Non-DPP-4 i	Reference		Reference	
DPP-4 i	0.51 (0.24–1.08)	0.078	0.49 (0.21–1.13)	0.094
Age (years)				
<40	Reference		Reference	
40–64	0.69 (0.21–2.29)	0.539	0.67 (0.19–2.36)	0.528
≥65	0.24 (0.06–1.01)	0.052	0.16 (0.03–0.79)	0.024
Sex				
Female	Reference		Reference	
Male	1.00 (0.48–2.07)	0.998	0.62 (0.28–1.37)	0.237
Biguanides	1.18 (0.56–2.47)	0.670	1.24 (0.52–2.96)	0.626
Glinides	1.28 (0.45–3.67)	0.647	1.65 (0.51–5.28)	0.400
Alpha-glucosidase inhibitors	0.62 (0.19–2.03)	0.424	0.76 (0.21–2.71)	0.671
Sulfonylurea	0.63 (0.30–1.29)	0.203	0.70 (0.30–1.64)	0.415
Thiazolidinediones	1.52 (0.62–3.73)	0.356	2.10 (0.80–5.53)	0.134
Insulin	1.09 (0.53–2.23)	0.819	0.93 (0.44–1.97)	0.846
Hypertension	1.64 (0.75–3.58)	0.214	2.72 (1.19–6.21)	0.018
Hyperlipidemia	0.78 (0.38–1.60)	0.490	0.57 (0.26–1.25)	0.161
Chronic liver disease	2.13 (0.97–4.64)	0.058	1.95 (0.87–4.40)	0.107
Chronic kidney disease	0.36 (0.05–2.63)	0.313	0.27 (0.03–2.18)	0.218
COPD	1.12 (0.27–4.71)	0.876	1.76 (0.39–8.04)	0.463
Malignancy	1.51 (0.46–4.98)	0.497	2.09 (0.60–7.27)	0.246
Stroke/TIA	0.50 (0.07–3.65)	0.491	0.46 (0.06–3.65)	0.463
Ischemic heart disease	0.65 (0.20–2.14)	0.480	0.67 (0.19–2.29)	0.521

TIA, transient ischemic stroke; COPD, chronic obstructive pulmonary disease.

## Discussion

In this large nationwide retrospective cohort study, we analyzed the risks of hospitalization for acute pancreatitis associated with DPP-4 inhibitors compared with non-DPP-4 inhibitors in patients with T2D with a prior history of acute pancreatitis. We found no significant increased risk of recurrent acute pancreatitis associated with DPP-4 inhibitors. Although it is reasonable to recommend avoiding these drugs for individuals with a history of pancreatitis, it is important to note that solid data on the real-world risk of recurrent pancreatitis are scarce. This is especially true for patients who have risk factors for developing pancreatitis. The initial disclosure of acute pancreatitis cases linked to exenatide and sitagliptin (or sitagliptin/metformin) made by the US FDA. The US FDA issued its first safety alert regarding acute pancreatitis and exenatide on 16 October 2007. This alert was based on reports of cases of acute pancreatitis among individuals using exenatide. The US FDA issued its first safety communication regarding acute pancreatitis and sitagliptin and sitagliptin/metformin on 25 February 2011. This communication was prompted by reports of pancreatitis in patients taking these medications. These announcements were made to inform healthcare professionals about potential risks associated with these drugs and to encourage monitoring and reporting of adverse events related to acute pancreatitis. Subsequent investigations and studies have aimed to assess the risk of acute pancreatitis associated with these medications in more detail.

Indeed, the literature on the association between drugs like DPP-4 inhibitors and acute pancreatitis can be complex and sometimes contradictory. Randomized clinical trials (RCTs) and observational studies often provide valuable insights into the safety and efficacy of medications, but their findings can vary due to differences in the study design, patient populations, and other factors. The results of a meta-analysis which pooled data from 134 RCTs suggest that the use of DPP-4 inhibitors is not significantly associated with an increased risk of acute pancreatitis (Nachnani et al., 2010). The reported odds ratio (OR) of 0.93 with a 95% confidence interval (CI) of 0.51–1.69 indicates that there is no statistically significant increase in the risk of pancreatitis associated with DPP-4 inhibitors based on the available RCT data. Additional systematic reviews encompassing both randomized and non-randomized studies have similarly indicated that incretin therapy, that is, DPP-4 inhibitors, does not seem to be correlated with an elevated risk of pancreatitis in individuals with T2D (Li et al., 2014). RCTs often have strict inclusion and exclusion criteria that may limit the representation of specific patient populations. As a result, RCTs may not always include a sufficient number of patients with T2D who are at a high risk of acute pancreatitis or who have significant comorbidities. Thus, the safety of DPP-4 inhibitors, or any medication, in specific subgroups of patients, such as those at high risk of acute pancreatitis, should be a subject of further study.

The relative risk of acute pancreatitis was significantly increased in diabetic patients compared to non-diabetic controls. This finding suggests that the underlying condition of diabetes itself may be a contributing factor to the risk of acute pancreatitis. Patients with a history of previous pancreatic disease had a substantially higher risk of acute pancreatitis than those without such a history (Green et al., 2015). This highlights the importance of considering pre-existing pancreatic conditions as a significant risk factor. Patients with hypertriglyceridemia also had an increased risk of acute pancreatitis (RR: 1.4). Increased levels of triglycerides are recognized to be linked with a heightened risk of pancreatitis, underscoring the significance of managing confounding factors such as the severity of diabetes and other risk factors for acute pancreatitis. In a nested case–control study analyzing Taiwan NHI data, Chou et al. documented a noteworthy escalation in the risk of acute pancreatitis among users of DPP-4 inhibitors with hypertriglyceridemia (adjusted OR: 1.80; 95% CI: 1.26–2.56) and pancreatic disease (adjusted OR: 17.29; 95% CI: 10.60–28.19) compared to non-users; however, the non-user group comprised a diverse set of patients receiving various anti-diabetic agents (Chou et al., 2014). The percentage of a prior hospitalization history for acute pancreatitis included in this study was only around 6%–8%. A recent case–control study conducted using an Italian administrative population-based database, which compared 1,003 cases admitted to the hospital for acute pancreatitis with 4,012 matched controls, revealed no heightened risk associated with incretin therapy (Chou et al., 2014). Given the inconsistent associations observed in prior studies, our study uniquely focused on individuals with T2D with a history of prior hospitalization for acute pancreatitis.

The findings from rodent models regarding the effects of exenatide and sitagliptin on pancreatic inflammation and neoplasia are indeed complex and can appear contradictory. Some rodent studies have suggested that exenatide and sitagliptin may increase inflammation in pancreatic acinar cells and promote the formation of intraepithelial neoplasia. This raises concerns about the potential adverse effects of these drugs on the pancreas (Rouse et al., 2014). Another study reported that exenatide improved the outcome of chemically induced pancreatitis (Tatarkiewicz et al., 2010). The effects of medications can vary based on the specific context of the study, including the animal model used, the dose of the medication, and the duration of exposure. Different rodent models may respond differently to these medications, which can explain the variation in findings. Translating findings from rodent studies to humans can be challenging. Rodent physiology and drug metabolism can differ significantly from that of humans.

Real-world evidence and observational studies that include diverse patient populations, including those with comorbidities, can complement RCT findings by providing insights into how drugs perform in more varied clinical settings. Additionally, post-marketing surveillance, ongoing monitoring, and pharmacovigilance efforts are essential for identifying and assessing rare or long-term adverse events associated with medications, even after they are approved for use. Research in specific patient subgroups can help refine our understanding of the safety and efficacy of medications like DPP-4 inhibitors.

This study’s primary strength lies in its inclusion of an extensive nationwide cohort of diabetic patients in Asian population. Presently, it stands as the largest observational study investigating the potential link between DPP-4 inhibitors and acute pancreatitis, uniquely concentrating on individuals with T2D, particularly those with a history of prior hospitalization for acute pancreatitis. The substantial number of incident cases of acute pancreatitis provides ample statistical power to elucidate any potential association. Second, this study included all DPP-4 inhibitors approved by the FDA, in contrary to previous studies which may not be generalizable to all DPP-4 inhibitors. However, several inherent limitations of observational studies need acknowledgment. Despite thorough adjustment using propensity scores, residual confounding factors cannot be entirely ruled out. Specifically, information on other risk factors like alcohol consumption, smoking, and obesity was lacking. Second, whereas the follow-up duration in this study is adequate for detecting acute pancreatitis development, subclinical low-grade pancreatic inflammation or non-hospitalized pancreatitis attacks may go undetected. In summary, based on this extensive nationwide cohort study in Taiwan with a significant number of incident cases, the use of DPP-4 inhibitors appears to be unrelated to an increased risk of recurrent acute pancreatitis, in those who have a prior history of acute pancreatitis in stable condition.

## Data Availability

The raw data supporting the conclusion of this article will be made available by the authors, without undue reservation.
