# Supplementary material for: Association of dipeptidyl peptidase-4 inhibitor and recurrent pancreatitis risk among patients with type 2 diabetes: A retrospective cohort study
Source: Front Pharmacol. 2024 Jul 4;15:1341363. doi: 10.3389/fphar.2024.1341363 (PMC11256048; doi:10.3389/fphar.2024.1341363)
Supplement: Supplementary file 1 [file Table1.docx]

Supplementary appendix

ICD Codes

Diabetes mellitus 250, E10, E11,E12,E13,E14

Type 2 DM (250, E11,E13, excluding 250.01,250.03,250.11,250.13

,250.21,250.23,250.31,250.33,250.41,250.43,250.51,250.53,250.61

,250.63,250.71,250.73,250.81,250.83,250.91,250.93,E10)

Type 2 DM (250, E11,E13, excluding 250.x1,250.x3,E10)

DM medications

dg25:DPP4 A10BH

dg26:Biguanides A10BA

dg27:Glinides A10BX02, A10BX03

dg28:Alpha-glucosidase inhibitors A10BF

dg29:Sulfonylurea A10BB

dg30:Thiazolidinediones A10BG02, A10BG03

dg31:Insulin A10A

dg52 GLP1 A10BJ

dg53 SGLT2 A10BK

Acute pancreatitis 577.0 B25.2 K85

K80 Cholelithiasis

574 Cholelithiasis

571.0 Alcoholic fatty liver

571.1 Acute alcoholic hepatitis

571.2 Alcoholic cirrhosis of liver

571.3 Alcoholic liver damage, unspecified

K70 Alcoholic liver disease

272.1 Pure hyperglyceridemia

E78.1 Pure hyperglyceridemia
